# Changes in temporal lobe activation during a sound stimulation task in patients with sensorineural tinnitus: a multi-channel near-infrared spectroscopy study

**DOI:** 10.1186/s12938-024-01255-7

**Published:** 2024-06-21

**Authors:** Xiaoli Fan, Bin Gong, Hao Yang, Juanjuan Yang, Gaowei Qi, Zheng Wang, Jie Sun, Yu Fang

**Affiliations:** 1 Department of Traditional Chinese Medicine, Songjiang Hospital Affiliated to Shanghai Jiao Tong University School of Medicine, Shanghai, 201699 China; 2 School of Mechanical and Automotive Engineering, Shanghai University of Engineering Science, Shanghai, 201620 China

**Keywords:** Sensorineural tinnitus, Functional near-infrared spectroscopy, Temporal lobe, Sound stimulation

## Abstract

**Background:**

The subjective sign of a serious pandemic in human work and life is mathematical neural tinnitus. fNIRS (functional near-infrared spectroscopy) is a new non-invasive brain imaging technology for studying the neurological activity of the human cerebral cortex. It is based on neural coupling effects. This research uses the fNIRS approach to detect differences in the neurological activity of the cerebral skin in the sound stimulation mission in order to better discriminate between the sensational neurological tinnitus.

**Methods:**

In the fNIRS brain imaging method, 14 sensorineural tinnitus sufferers and 14 healthy controls listened to varied noise and quiet for fNIRS data collection. Linear fitting was employed in MATLAB to eliminate slow drifts during preprocessing and event-related design analysis. The false discovery rate (FDR) procedure was applied in IBM SPSS Statistics 26.0 to control the false positive rate in multiple comparison analyses.

**Results:**

When the ill group and the healthy control group were stimulated by pink noise, there was a significant difference in blood oxygen concentration (*P* < 0.05), and the healthy control group exhibited a high activation, according to the fNIRS measurement data. The blood oxygen concentration level in the patient group was dramatically enhanced after one month of acupuncture therapy under the identical stimulation task settings, and it was favorably connected with the levels of THI and TEQ scales.

**Conclusions:**

Using sensorineural tinnitus illness as an example, fNIRS technology has the potential to disclose future pathological study on subjective diseases throughout time. Other clinical disorders involving the temporal lobe and adjacent brain areas may also be examined, in addition to tinnitus-related brain alterations.

## Introduction

The most prevalent clinical symptom in the clinical categorization of tinnitus is sensorineural tinnitus, often known as subjective tinnitus [1]. Tinnitus symptoms affect more than 10% of the world's population, according to data [2]. Tinnitus is frequently associated with deafness, and it can progress to deafness. Both might emerge at the same time or in a different order [3]. According to clinical statistics, there are presently more than 50 million individuals in China over the age of 50 who suffer from deafness and tinnitus, and 30 percent to 50 percent of the elderly over the age of 60 suffer from deafness and tinnitus [4]. Deafness and tinnitus have become a significant element that has a negative impact on middle-aged and older people's quality of life, as well as family harmony and happiness.

Although animal models of tinnitus have been reported, tinnitus is, after all, a subjective symptom of sufferers [5]. Because there are currently few research techniques that can objectively detect the presence of tinnitus, objectively measuring the therapeutic efficacy of a particular treatment strategy on tinnitus is impossible. Functional near-infrared spectroscopy (fNIRS), a new non-invasive neuroimaging method that can quantify the degree of neural activity in the human brain through hemodynamic rate and resting-state functional connectivity, has been popular in recent years [6]. In addition, when compared to other brain function imaging technologies, fNIRS is characterized by features such as non-invasiveness, broad applicability to diverse populations, relatively high temporal resolution, and strong robustness, thus being widely applied in brain function detection [7, 8].

In 1977, Jobsis first detected brain activity optically, confirming the feasibility of using radiative spectra to probe the brain [9]. The linkage between near-infrared signals and cerebral oxygenation was established by Delpy et al., who defined the modified Beer–Lambert law, significantly advancing the detection of brain activity [10]. The activation of the cerebral cortex during wrist movement was assessed using functional near-infrared spectroscopy by Jalalvandi et al. [11]. Using near-infrared spectroscopy, Sun et al. revealed reduced prefrontal activity during a verbal fluency challenge in individuals with chronic insomnia problem [12]. Martin Schecklmann and colleagues employed functional near-infrared spectroscopy to diagnose the status and symptoms of persistent tinnitus sufferers [13]. Basura Gregory et al. measured cochlear implant performance and tinnitus perception using functional near-infrared spectroscopy [14]. To enhance the possibilities of human brain imaging, Zhai Tianqu et al. used near-infrared spectroscopy to detect tinnitus and auditory cortex [15]. These papers show that functional near-infrared spectroscopy has been tried as a technological tool for determining the severity of disease.

This study aims to utilize fNIRS technology to observe changes in temporal lobe oxyhemoglobin concentration and brain region activation responses during specific sound stimulus tasks in both healthy individuals and patients with subjective tinnitus. Following comparative experiments, acupuncture treatment was administered to tinnitus patients for one month by specialized traditional Chinese medicine practitioners, with changes in temporal lobe oxyhemoglobin concentration during sound stimulus tasks being monitored. By applying fNIRS methodology, differences in cortical neural activity between patients with sensorineural tinnitus and healthy controls during sound stimulus tasks are sought to be discerned, aiming to enhance the distinction of sensorineural tinnitus symptoms. Leveraging sensorineural tinnitus as a focal point, promise is held by fNIRS technology in gradually unraveling future pathophysiological investigations into subjective disorders. Beyond investigating cerebral alterations associated with tinnitus, other pathological states involving the temporal lobe and surrounding brain regions may also be explored.

## Results

### Characteristics of population sample

Table 1 shows the characteristics of the test individuals in the sample. Between the healthy control group and the sensorineural tinnitus patients, there were statistically significant differences in THI, TEQ, HAMD, and HAMA scores (*P* < 0.05). As expected, scale scores were significantly higher in the sensorineural tinnitus group than in the control group. However, there was no statistically significant change in the scores of the scales before and after one month of therapy for patients with sensorineural tinnitus (*P* > 0.05).

**Table 1:** Sample characteristics

Demographics	Patient group	Healthy control group	Group difference
Age [years]	48 ± 11.68	26.86 ± 3.60	*P* = 0.000
Sex [female/male]	10/4	13/1	*P* = 0.139
THI	44.43 ± 28.16	–	–
TEQ	10.15 ± 5.02	–	–
HAMD	15.93 ± 11.24	3.79 ± 8.51	*P* = 0.003
HAMA	14.43 ± 10.37	5.43 ± 8.49	*P* = 0.019

Data expressed as mean ± standard deviation or n/n

### fNIRS analysis

**Fig. 1:**
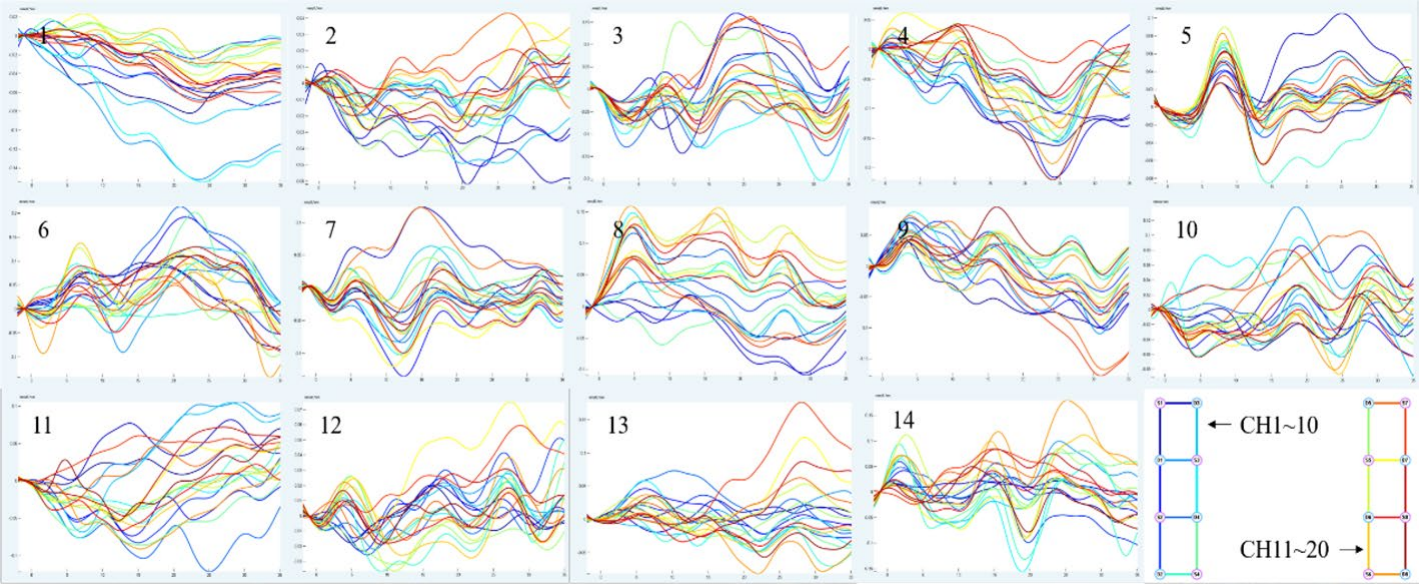
Healthy control group pink noise stimulus

**Fig. 2:**
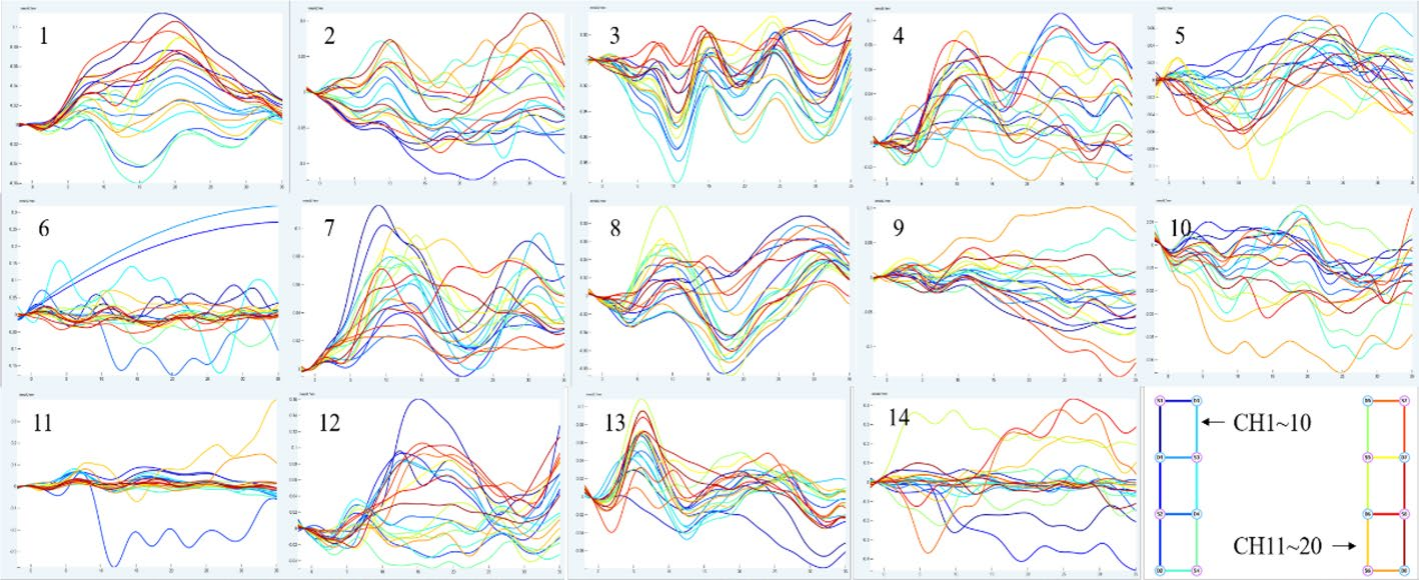
Patient group pink noise stimulus

**Fig. 3:**
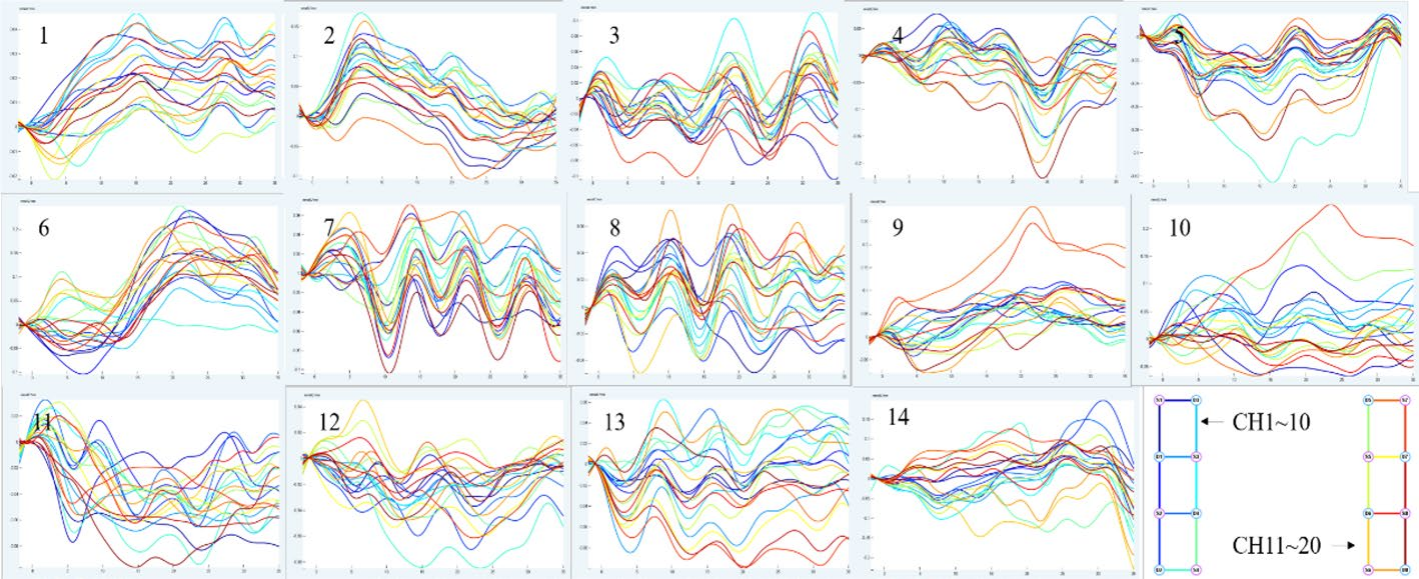
Healthy control group 750 Hz noise stimulus

**Fig. 4:**
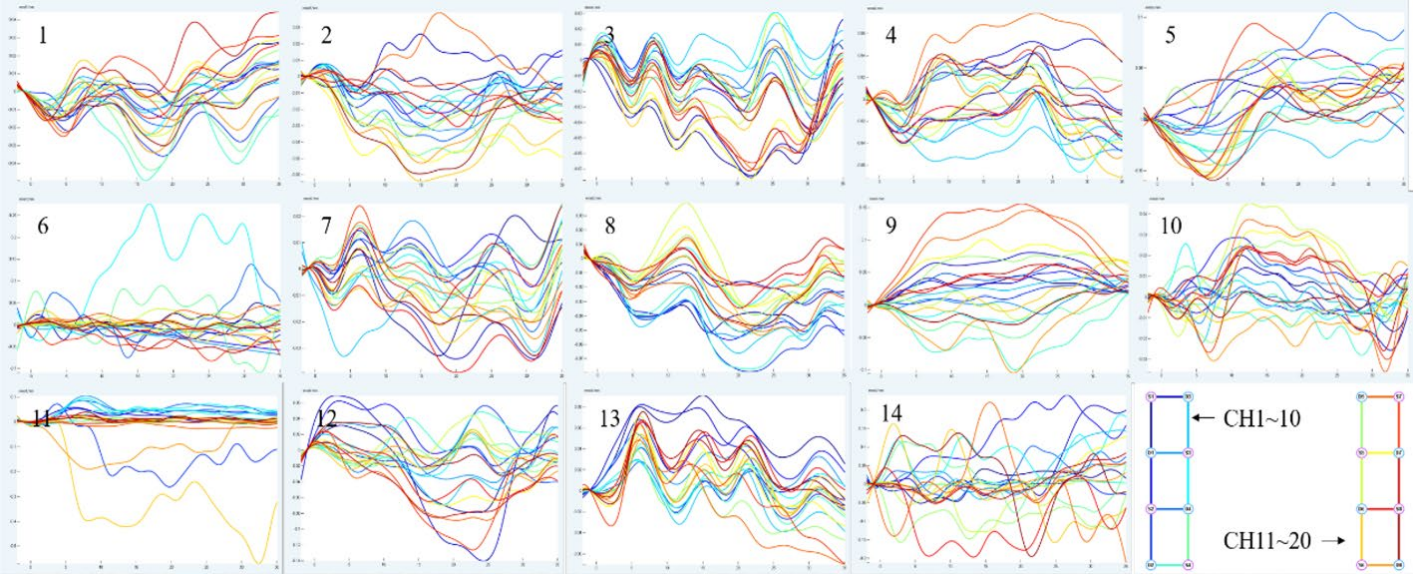
Patient group 750 Hz noise stimulus

**Fig. 5:**
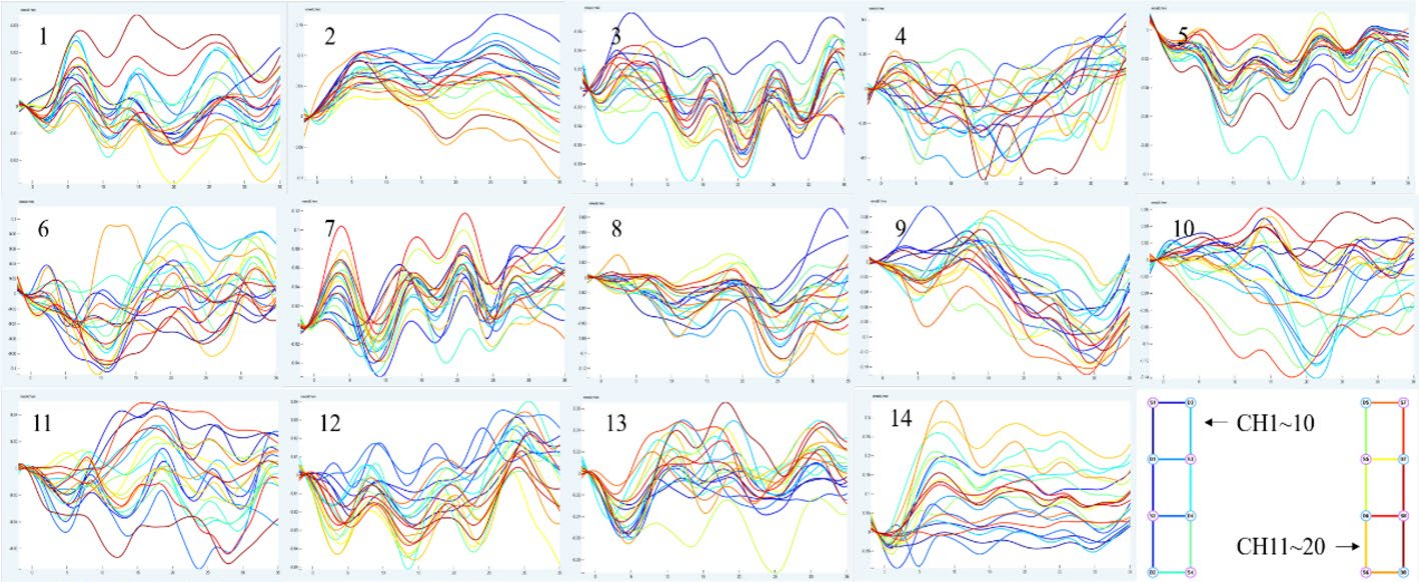
Healthy control group 8000 Hz noise stimulus

**Fig. 6:**
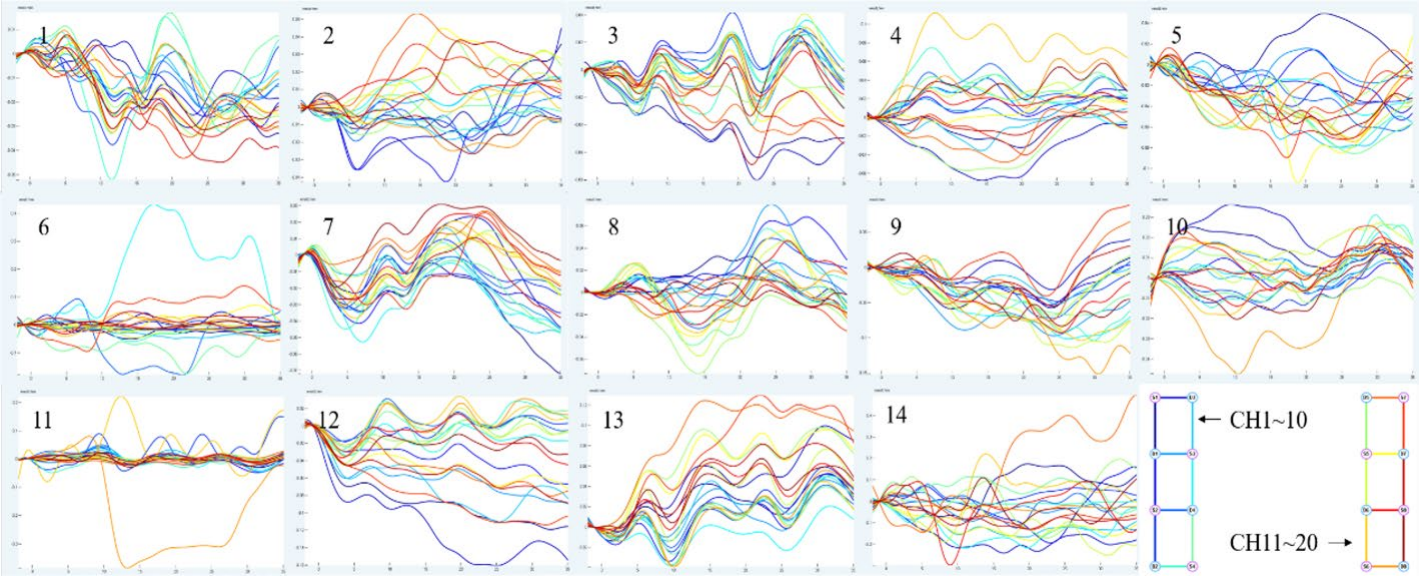
Patient group 8000 Hz noise stimulus

Figure 1 depicts the mean data curve of oxyhemoglobin block in the temporal lobe of healthy people activated by pink noise. Figure 2 depicts the mean data curve of oxyhemoglobin block in the temporal lobe of patients who were stimulated by pink noise. The average data curve of oxyhemoglobin block in the temporal lobe of the healthy group under the stimulation of 750 Hz noise is shown in Fig. 3. Similarly, the mean data curve of oxyhemoglobin block in the temporal lobe of the patient group under 750 Hz noise stimulation is shown in Fig. 4. The mean data curve of oxyhemoglobin block in the temporal lobe of the healthy group under noise stimulation at 8000 Hz is shown in Fig. 5. Figure 6 shows the mean data curve of oxyhemoglobin block in the temporal lobe of the patient group during 8000 Hz noise stimulation.

The average oxyhemoglobin block data acquired above were then imported into IBM SPSS Statistics 26.0 for independent sample *T*-test analysis, followed by the group statistical analysis stage. The eigenvalue was selected as the difference value, that is, the mean difference between the time interval between 10 ~ 15 s and 30 ~ 35 s after the stimulation started. The FDR correction was done at the same time as the two-tailed test. The hypothesis test has a significance level of *P* < 0.05.

**Fig. 7:**
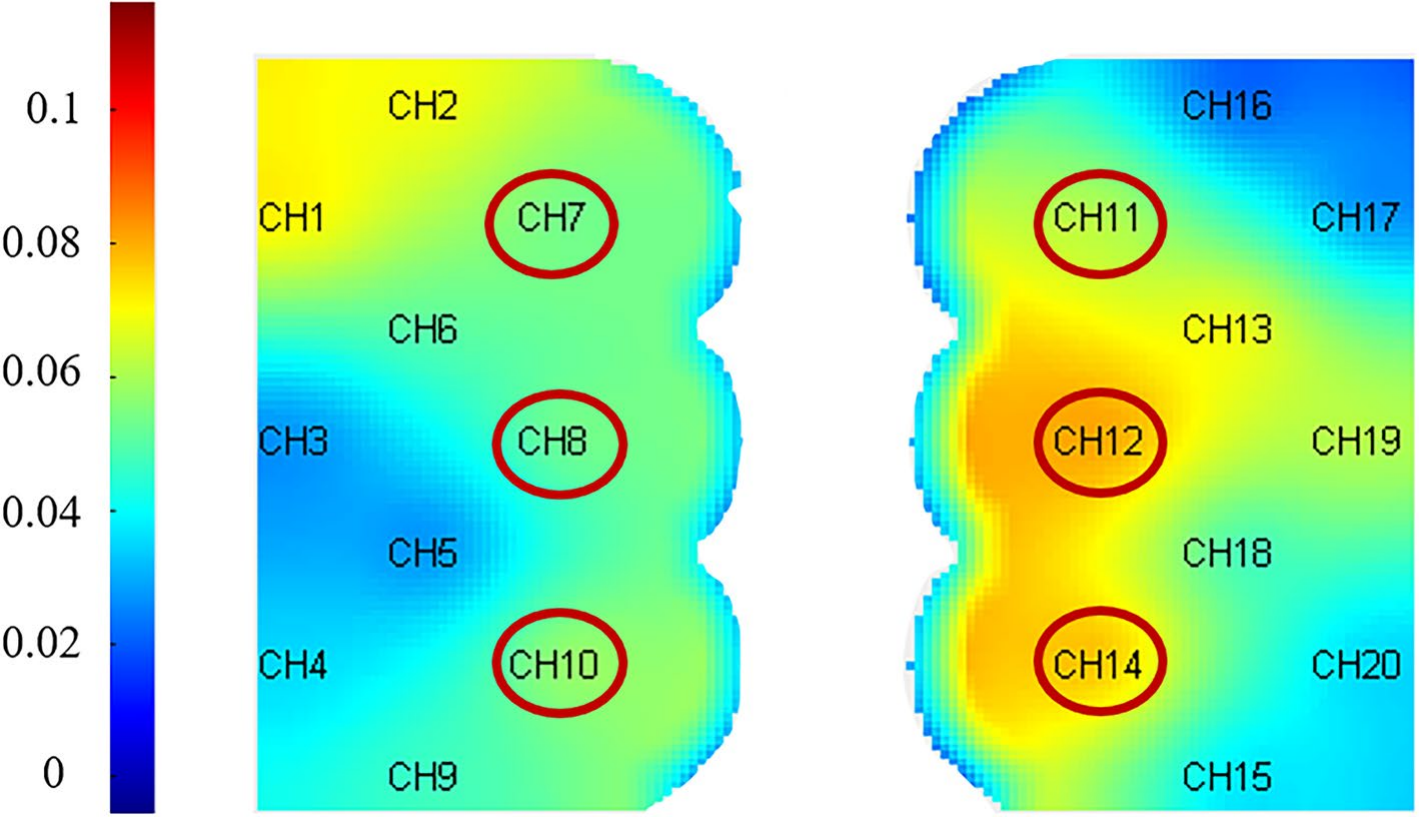
Schematic diagram of significant channel

The three types of stimulus noise were compared using fNIRS data. Under pink noise stimulation, there was a significant difference in blood oxygen concentration between the two groups (*P* < 0.05), as well as a significant negative connection between changes in oxygenated hemoglobin level and THI and TEQ scores. From the overall data of the 20 channels, the oxyhemoglobin trends of CH7-8, CH10-12 and CH14 channels near the parietal lobe region showed significant fluctuations. The mean concentrations of oxyhemoglobin across the 20 channels recorded at a certain moment for both tinnitus patients and healthy control groups are documented in Table 2. On the whole, lower oxyhemoglobin concentrations were observed in the healthy control group compared to the diseased group, with key channels depicted in Fig. 7. Finally, blood oxygen concentration levels were considerably higher after one month of acupuncture therapy under the identical stimulation task settings, and were favorably linked with THI and TEQ scale levels.

**Table 2:** The average concentrations of oxygenated hemoglobin across 20 channels for patients with sensorineural tinnitus and normal person

Serial number	Oxyhemoglobin concentration	
	Patient	Normal person
1	0.03930815	− 0.04128
2	0.0098162	− 0.0211
3	0.0445445	− 0.00431
4	0.022166	− 0.03778
5	0.00081665	0.0254
6	− 0.03452	− 0.03919
7	− 0.0075	− 0.01948
8	0.032394	0.004043
9	− 0.00985	− 0.02543
10	− 0.01155	− 0.00887
11	0.029762	0.03378
12	− 0.03952	0.016016
13	0.009431	0.000252
14	0.012653	− 0.01779

## Discussion

The study combined fNIRS technology with a stimulation task for patients with sensorineural tinnitus, aiming to detect changes in temporal lobe activation and better distinguish the symptoms of sensorineural tinnitus. fNIRS signals were collected from 14 sensorineural tinnitus patients and 14 healthy controls under different noise conditions, and the changes in oxygenated hemoglobin concentration in sensorineural tinnitus patients were analyzed.

The collected fNIRS data during the measurement process are in the form of regional averages rather than absolute quantitative values. If changes occur at a rapid rate throughout the entire measurement process, the accuracy of experimental results may be compromised [16]. However, these data can still indirectly reflect local brain activity. The regional averaging approach constrains the precision of measurements, yet fNIRS, as a non-invasive and user-friendly brain imaging technique, retains significant applications across various domains. Improvements in hardware and algorithms in the future may enhance measurement precision.

The data collection in this study encompassed only 14 patients with sensorineural tinnitus and 14 healthy controls, potentially introducing bias into the results. Adequate sample size or participant numbers are paramount for any near-infrared spectroscopy experiment, yet there exist no fixed rules to ensure statistical validity [17]. Despite efforts to minimize errors, further research is imperative to determine the optimal sample size for this experiment, thus ensuring accurate representation of broader research findings.

fNIRS, as a brain functional imaging technique based on hemodynamic changes, is quiet and has relatively good resistance to motion interference, making it more suitable for tinnitus research than other imaging methods. However, fNIRS can only measure the hemodynamic activity of the superficial cortical regions, and not the deep brain activity [18]. In this study, although all participants had sensorineural tinnitus, the acupuncture treatment received in the last month may have been influenced by other physical and mental factors, leading to uncertainties in the result analysis. Future research may consider adopting a more rigorous experimental design and conducting more detailed measurement and statistical adjustments of potential confounding factors.

## Conclusions

As fNIRS technology advances, this approach will become increasingly useful in studying subjective diseases in humans. In this work, sensorineural tinnitus illness was used as a starting point, and a sound stimulation task was used to determine that under pink noise stimulation, fNIRS data from the temporal lobe indicated a significant difference between the patient and control groups. These findings might reveal new information about the pathophysiology of sensorineural tinnitus, and this novel and highly inventive way to imaging the brain using fNIRS technology could give a more comprehensive and non-invasive approach. The widespread use of fNIRS to examine central auditory circuits in humans will help us better understand the normal and aberrant circuits that exist after hearing loss and tinnitus in the future.

## Materials and methods

### Subject of the experiment

In this study, 14 patients with sensorineural tinnitus (10 men and 4 females) were recruited and selected among the outpatients of the Central Hospital of Songjiang District in Shanghai, ranging in age from 27 to 67 years. The following were the criteria for inclusion: (1) those between the ages of 18 and 75; (2) those who presented with tinnitus as their primary complaint and were diagnosed as having subjective tinnitus; (3) those who fully comprehended the tinnitus handicap inventory (THI), tinnitus evaluation questionnaire (TEQ), Hamilton Depression Scale (HAMD), and Hamilton Anxiety Scale (HAMA); (4) those who were able to complete the fNIRS test; (5) the specialized examination revealed no external ear disease or acute inflammatory disease of the middle ear [19].

Both the sick group and the healthy control group met the following selection criteria in this experimental study: (1) all subjects were right-handed; (2) all subjects were native Chinese speakers; (3) all subjects had normal color vision and eyesight; (4) in the first three days of the experiment, there was no significant quantity of rigorous exercise or significant mood swings [20]. Before the commencement of the trial, the 28 subjects were told about the nature of the trial and its safety concerns, and they signed an informed consent form for the trial's implementation that met the ethical committee's requirements. And, guided by the Helsinki Declaration, to protect their legitimate rights and interests [21].

### Clinical assessment (case report form)

The subjects filled out the case report form with the help of a competent doctor, and the degree of sensorineural tinnitus was determined by the THI and TEQ [22]. The HAMD and HAMA were added to the case report form in order to assess the presence and severity of depressive and anxiety symptoms in the subjects [23, 24].

### Sound stimulation task

**Fig. 8:**
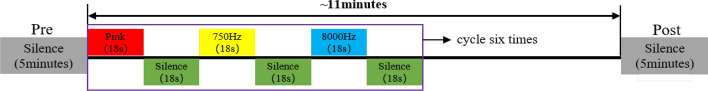
fNIRS record paradigm

Throughout the test, the subjects wore noise-cancelling headphones to guarantee that they were not bothered by the surrounding surroundings [25]. Figure 8 depicts the passive listening block paradigm design plan for the sound stimulus task. There are three auditory stimulus blocks in total: 18 s of pink noise with 1/f-characteristics (It was frequently used to facilitate synchronization of neural oscillatory activities), 18 s of 750 Hz noise, and 18 s of 8000 Hz noise [26]. Following that, each auditory stimulus block was alternated with 18-s quiet blocks that were played in six cycles for around 11 min [27]. The sound pressure level of all auditory stimulus blocks was controlled at 50 dB, and a 5-min silence interval was set before and after the use of the paradigm to calculate the resting-state brain functional connection [28].

### fNIRS measurement

**Fig. 9:**
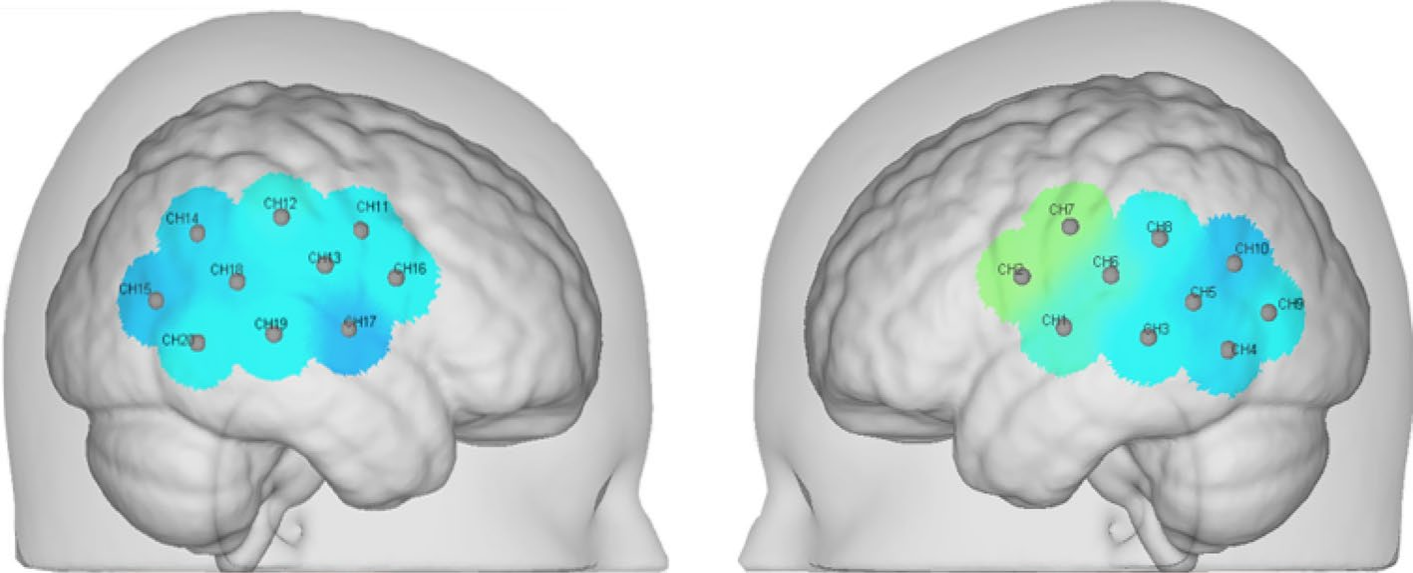
Temporal lobe measurement channel layout

Because it is necessary to work back and forth between hospitals and universities during the data collection process, and in order to reduce measurement results caused by equipment errors during the data collection process, the near-infrared spectrum functional imaging equipment used in this article must have extremely high portability and robustness [29]. As a result, Huichuang's independent model, the NirSmartII-3000c near-infrared brain function imaging system, is used in this study [30]. This gadget satisfies all of the test conditions outlined in this article. During the acoustic stimulus task, the relative concentrations of oxyhemoglobin, deoxyhemoglobin, and total hemoglobin were assessed using a modified Beer–Lambert equation [31, 32]. The temporal lobe, according to the requirements of Brodmann division, is primarily responsible for language and auditory perception, as well as long-term memory and emotion [33]. To evaluate bilateral temporal lobe areas of the brain, a total of 20 channels (CH1–CH20) were established for fNIRS signal collection [34]. The emission bands of near-infrared light were 730 nm and 850 nm, and the sampling rate was set to 20 Hz [35]. Figure 9 depicts the channel configuration for the 20 channels.

### Statistical analysis

**Fig. 10:**
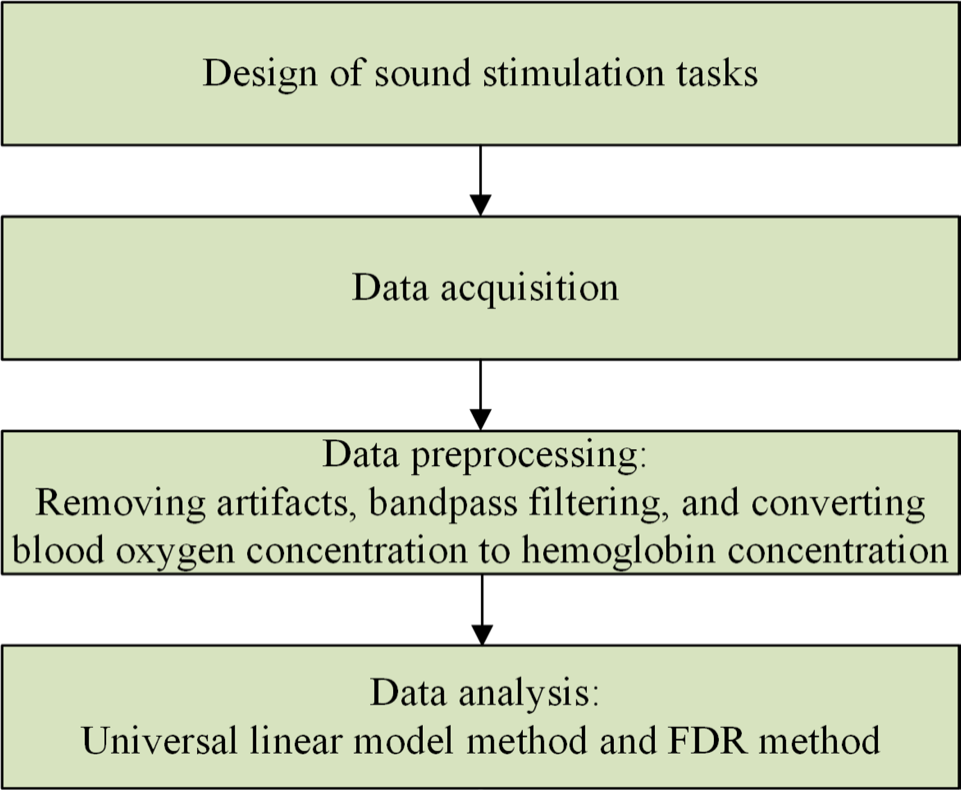
Data flowchart in near-infrared spectroscopy research

Except for the Chi-square test for gender, a two-sample *T*-test was employed to compare the average values between sensorineural tinnitus sufferers and healthy controls in this study's examination of sample characteristics and clinical evaluation [36]. Before statistical analysis of collected fNIRS data, the data must first go through a series of pretreatments, which include deleting unrelated intervals from the experiment, eliminating artifacts that have nothing to do with the experimental data, and filtering the data with low-pass and high-pass filters, and finally calculating the ultimate oxygenated hemoglobin concentration data using blood oxygen [37]. The mean value of the first 2 s of the auditory stimulus and the mean value of the 35-s interval after the stimulus were calculated for the block design measurement analysis of the stimulus task to remove the gradual drift in the measurement [38]. The average trajectory for each stimulus was derived by averaging six repeats. The goal of calculating block average is to use superposition average to remove the random noise from each trial of the same task, resulting in the task's average blood oxygen response function [39]. We utilized the average oxygenation amplitude during the stimulation for statistical analysis. The sluggish drift in the signal is minimized in event-dependent designs by utilizing a high-pass filter with six discrete cosine basis functions [40]. We used the general linear model method to do the analysis on MATLAB (MathWorks, USA) through a homemade script [41]. Statistical analysis software was IBM SPSS Statistics 26.0 (SPSS Inc, USA) [42]. A false detection rate (FDR) approach was employed for the repeated comparison analysis of the 20-channel fNIRS data to verify that no more than 5% of false positives were found on average [43]. The data flow diagram is illustrated in Fig. 10.

## Data Availability

The data that supported the findings of the present work are available from the corresponding author upon reasonable request.
